# The Motives Proper to the Physician

**Published:** 1909-08-14

**Authors:** 


### The Motives Proper to the Physician.

If the practice of medicine is not to sink in the regard of all concerned to the position of a mere means of livelihood, its votaries must possess a sufficiency of idealism. To promote this state of affairs is one great object, we take it, of all modern successors to and substitutes for the Hippocratic oath, pledges and admonitions innumerable to which about the latest addition is Professor Cleland's farewell address* to this year's medical graduates of Glasgow University. Wisely enough, the Professor addresses himself not to the flower of his class, but, to use the popular, ungrammatical phrase, to the average man; and his philosophy is much after the style of the stoicism of Marcus Aurelius. You cannot, perhaps, all be brilliant, he says, but you can all be good. To be really good means to most people a struggle—self-abnegation and so forth—and, therefore, Burns' (advised) "firm resolve . . . stalk of carl-hemp in man" is to be earnestly striven after. What constitutes being really good is to give hearty mutual service and thus to fulfil the natural law of the universe. In the application to the medical life some allowance is made for difficulties revealed by modern culture. "Resolution is a quality which, like every other feature, is largely dependent upon heredity, and heredity is not of our own making." Not far off the reader finds it stated that in the medical profession resolution is especially required, whether to face the sights of the dissecting room or accident ward, to pronounce death sentences, or to refrain from giving placebos or playing to the gallery. Clearly the deduction is that the good doctor, to reverse Ben Jonson's aphorism on the good poet, is born as well as made. Again, there is the necessitarian view of the will to be considered. Altruism, perhaps, is what one may indicate as Professor Cleland's bridge over these obstacles. The motive of the necessary self-abnegation must be love. In all his work, the noblest type of physician is animated by love—whether of his patients or of noble scientific ends.

We should rather have said the happiness resulting from true discharge of function: and, indeed, here there might (for the address has the fault, the good fault, of being rather short) have well followed a contrast between the working of two great forces concerned in the production of disinterested, worthy medical practitioners. When members of the laity want to speak nicely of our profession they dwell on the beneficence of its work. There is a pleasant implication that the doctor is a man overflowing with the milk of human kindness; almost a suggestion that the best doctor is the most sympathetic one. This is hardly the esoteric view. Sympathy is a quality so greedily laid claim to by quacks as not to be in very high repute in the medical profession; some may remember the performance of a certain music-hall "doctor," who, his eyes rolling in anguish, would exclaim to an attentive audience that his work exhausted him terribly, but that his abundant consolation lay in the reflection that it was all for the good of others. Undoubtedly the best physicians and surgeons have been those who had naturally the objective and not the subjective outlook, whose pleasure in their work was artistic. Hence, perhaps, the success of the Abernethian manner, for patients, though much deceived at times, can generally recognise outstanding ability. And hence too, maybe, the impression—on a level with that of George Eliot's rustics, who considered it a mark of their lawyer Dempsey's undeniable capacity that he was a dissolute knave—of anti-vivisectionists that a skilful doctor is apt to be cold-blooded and ruthless. This melodramatic conception is much ministered to by the fiction of the cheaper magazines. Unfortunately, it is probable that in other ways as well the conscientious clinician's motives are often misinterpreted.

* *Remarks on Factors at Work in Life. A valedictory address to the Graduates in Medicine and Surgery in the University of Glasgow, July 13, 1909.* By John Cleland, M.D., LL.D., D.Sc., F.R.S., Regius Professor of Anatomy.

